# Modulating Room-Temperature Phosphorescence-To-Phosphorescence Mechanochromism by Halogen Exchange

**DOI:** 10.3389/fchem.2021.812593

**Published:** 2022-01-13

**Authors:** Yoshika Takewaki, Takuji Ogawa, Yosuke Tani

**Affiliations:** Department of Chemistry, Graduate School of Science, Osaka University, Toyonaka, Japan

**Keywords:** mechanochromism, heavy atom effects, isostructural crystals, amorphous, halogen exchange, room-temperature phosphorescence, metal-free

## Abstract

Modulating the stimulus-responsiveness of a luminescent crystal is challenging owing to the complex interdependent nature of its controlling factors, such as molecular structure, molecular conformation, crystal packing, optical properties, and amorphization behavior. Herein, we demonstrate a halogen-exchange approach that disentangles this problem, thereby realizing the modulation of room-temperature phosphorescence-to-phosphorescence mechanochromism. Replacing the bromine atoms in a brominated thienyl diketone with chlorine atoms afforded isostructural crystals; i.e., molecules with different halogen atoms exhibited the same molecular conformation and crystal packing. Consequently, amorphization behavior toward mechanical stimulation was also the same, and the phosphorescence of amorphous states originated from the same conformer of each diketone. In contrast, the phosphorescence properties of each conformer were modulated differently, which is ascribable to heavy atom effects, resulting in the modulation of the mechanochromism. Thus, halogen exchange is a promising approach for modulating the stimulus-responsive photofunctions of crystals involving spin-forbidden processes.

## Introduction

Mechanochromic luminescence is a phenomenon in which the luminescence color changes when mechanical stimulus is applied and is recovered by other external stimuli, such as heat (Sagara et al., 2016; Ito, 2021). Such phenomena have received significant interest not only because they visualize otherwise invisible force histories, but also because of their mechanism involved in transducing macroscopic force to the molecular level. Basically, the color change is due either to a change in the molecular environment, the intermolecular arrangement, the molecular conformation, or a combination thereof.

Phosphorescent organic molecules are particularly promising mechanochromic materials because phosphorescence is highly sensitive to changes in the molecular environment (Xue et al., 2016; Huang et al., 2020). Phosphorescence is a spin-forbidden form of luminescence that involves a change in the spin multiplicity. Unlike metal complexes, such as Ir and Pt, which benefit from the heavy atom effect that accelerates spin-forbidden processes, metal-free organic molecules seldom show room-temperature phosphorescence (RTP) (Hirata, 2017; Kenry et al., 2019). However, the crystalline states of some metal-free organic molecules were reported to exhibit RTP as early as 1939 (Clapp, 1939; Bilen et al., 1978). More recently, significant interest has been directed toward “crystallization-induced phosphorescence” (CIP) following the seminal report published in 2010 by Tang et al. (Yuan et al., 2010) A rigid environment is generally regarded to be crucial for observing RTP from a metal-free organic molecule; otherwise, RTP is quenched by molecular motions (Baroncini et al., 2017). Indeed, the conventional RTP of an organic crystal is quenched by applying mechanical stimulation, which amorphizes the crystal. While such mechanoresponsive RTP turn-off behavior has been used to achieve RTP-to-fluorescence mechanochromism, (Gong et al., 2015; Mao et al., 2015; Xu et al., 2017; He et al., 2019; Huang et al., 2019; Ma et al., 2019; Wang et al., 2019a; Liu et al., 2021a; Liu et al., 2021b) the RTP mechanoresponse is largely limited to the turn-off type, with a molecular design that modulates mechanochromic RTP behavior remaining elusive.

To establish design principles for mechanochromism, two types of structure–mechanochromic-property relationship are important: the molecular-structure–property and the crystal-structure–property relationship. However, in many cases, the molecular structure and crystal structure are strongly interdependent, which prevents revealing the essential causal relationship. Polymorphic crystals, in which the same molecule forms different crystal structures, provide practical bases for studying the crystal-structure–property relationship (Wang and Li, 2017). On the other hand, the molecular-structure–property relationship can be elucidated when different molecules form isostructural crystals; i.e., crystals with the same space group, lattice constants, crystal packing, and molecular conformations (Fábián and Kálmán, 1999; Kálmán et al., 1993). The differences in isostructural crystal structures are so small that they usually do not disturb the properties derived from the molecular structure itself.

**FIGURE 1:**
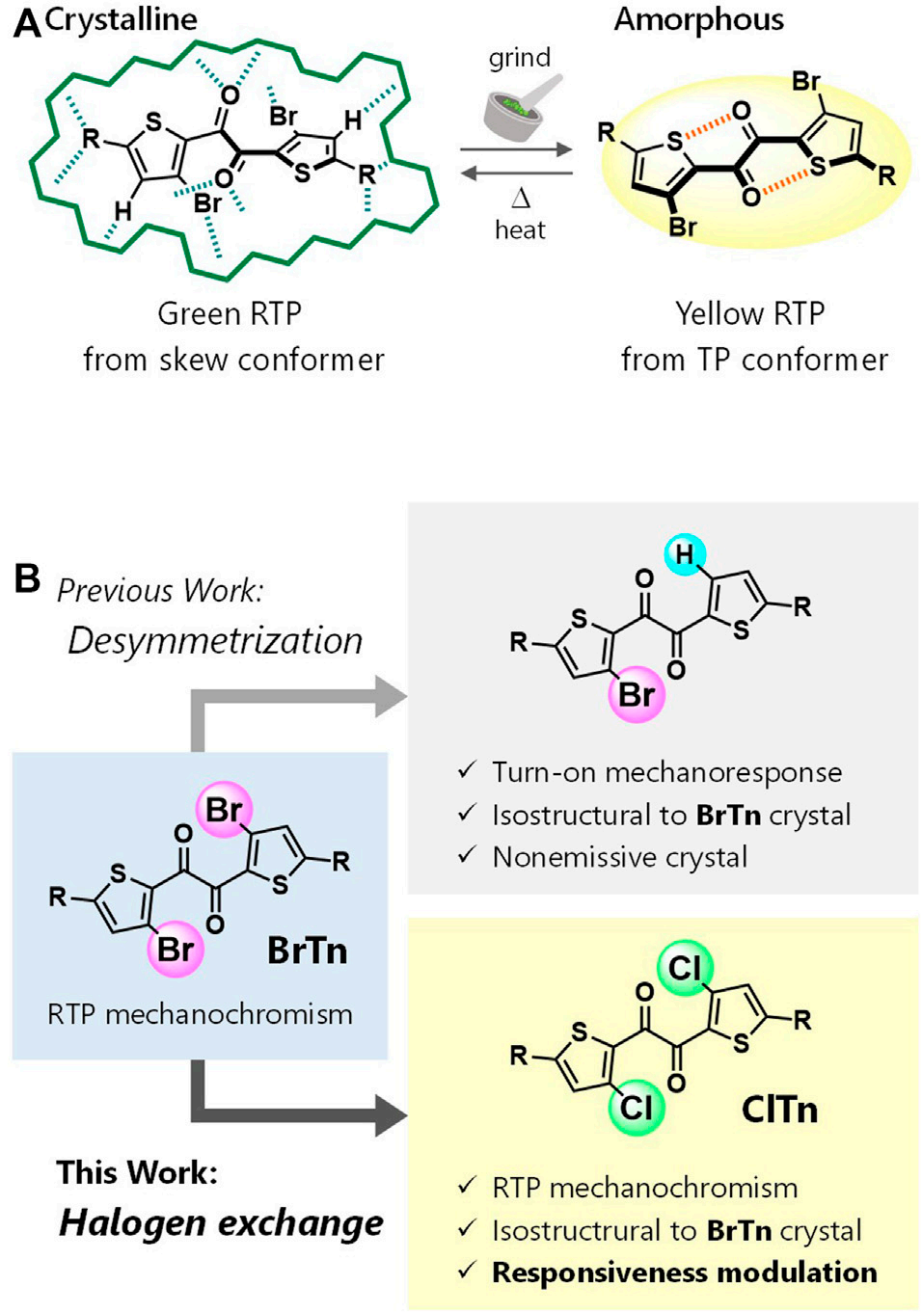
**(A)** Schematic depicting the RTP-to-RTP mechanochromism of **BrTn**. Green hashed lines represent intermolecular interactions, whereas orange ones represent intramolecular chalcogen bonds. **(B)** Molecular design for modulating mechanoresponsive RTP. R, triisopropylsilyl.

Previously, we reported the thienyl diketone **BrTn** (Figure 1A), the first metal-free organic molecule to exhibit RTP-to-RTP mechanochromism (Tani et al., 2019); in contrast to RTP quenching observed for conventional organic phosphors, mechanical stimulation amorphizes the **BrTn** crystals, resulting in RTP color change. Detailed investigations revealed that mechanical stimulus turns off the initial RTP (from a skew conformer) while providing a small amount of the highly emissive trans-planar (TP) conformer. In addition, we desymmetrized the *C*_2_-symmetrical **BrTn** structure. (Tani et al., 2020; Komura et al., 2021) Notably, replacing one of the two Br atoms with a hydrogen atom led to the formation of a crystal that is isostructural with that of **BrTn** (Tani et al., 2020). Interestingly, the crystal of the unsymmetrical diketone was non-emissive, which was attributed to the presence of voids that result from the volumetric difference between Br and H (Figure 1B top). Hence, the difference in the crystal structures were highly localized, facilitating the stringent crystal-structure–property relationship study.

Herein, we report the first RTP-to-RTP mechanochromism modulated by halogen-atom exchange. We envisaged that halogen-atom exchange in **BrTn** (e.g., bromine to chlorine) would afford isostructural crystals (Reddy et al., 2006; Saidykhan et al., 2021; He et al., 2017; Wen et al., 2019; Lai et al., 2021) that retained the mechanoresponsive nature of **BrTn**, as observed for our previous system (Figure 1B top). On the other hand, altering the heavy atoms is expected to affect the molecular phosphorescence properties, thereby modulating the responsiveness to RTP color. Heavy atom effect—the enhancement of the rate of a spin-forbidden process by the presence of an atom of high atomic number—has been widely used to modulate or achieve phosphorescence from metal-free organic molecules. (He et al., 2017; Wen et al., 2019; Lai et al., 2021; Wang et al., 2019b; Liao et al., 2021) With these aims in mind, we designed new diketone **ClTn**, in which the Br atoms in **BrTn** are replaced with Cl atoms (Figure 1B bottom). As a result, the fundamental stimulus-responsive nature of **BrTn** is retained, while the sensitivities of RTP color to mechanical/thermal stimuli are modulated. Our study demonstrates the usefulness of the halogen-exchange strategy for modulating the stimulus-responsive photofunctions of crystals that involve spin-forbidden processes.

## Experimental Section

### Instrumentation and Chemicals

^1^H and ^13^C{^1^H} NMR spectra were recorded on a JEOL ECS400 spectrometer. Chemical shift values (δ) are reported in ppm and are calibrated to tetramethylsilane (0.00 ppm) for ^1^H and to CDCl_3_ (77.0 ppm) for ^13^C NMR. Melting points were measured between cover glasses with Yanaco MP-S3. Elemental analysis (EA) was conducted on a Yanaco MT-6 recorder. Analytical thin-layer chromatography (TLC) was performed on aluminum plates bearing a layer of Merck silica gel 60 F_254_. Column chromatography was carried out on silica-gel 60N (Kanto Chemical Co., Inc., spherical, 63–210 μm). Unless otherwise noted, chemicals were obtained from commercial suppliers and used without further purification.

### Synthesis of ClTn

To a heat gun-dried Schlenk flask under Ar were added **BrTn** (69.9 mg, 0.101 mmol) (Tani et al., 2019), copper chloride (I) (50.2 mg, 0.507 mmol), and *N,N*-dimethylformamide/*o*-dichlorobenzene (3:1, 2.4 ml). The solution was degassed by typical freeze-pump-thaw cycling three times. Then, the mixture was stirred at 140°C overnight, quenched by adding aq. NH_4_Cl, and extracted with CHCl_3_ (10 ml × 3). The combined organic extracts were washed with aq. NH_4_Cl (10 ml × 2) and water (10 ml × 2), dried over MgSO_4_, and concentrated under reduced pressure. The crude product was passed through a silica-gel column (eluent: CHCl_3_), and all the volatiles were removed. The residue was further purified in refluxing MeOH (5 ml) at 75°C (bath temperature) for 20 min, and then filtered at room temperature. The solid was then suspended in hexane (2 ml) and refluxed at 80°C (bath temperature) for 20 min, cooled in a freezer, and filtered to give 34.5 mg (57.1 µmol, 57%) of **ClTn** as a yellowish-white powder. **m. p.** 156–158°C. ^**1**^ **H NMR** (400 MHz, CDCl_3_) δ: 7.17 (2H, s), 1.38 (6H, sep, *J* = 7.3 Hz), 1.12 (36H, d, *J* = 7.3 Hz). ^**13**^ **C NMR** (100 MHz, CDCl_3_) δ: 182.21, 148.75, 137.33, 134.64, 133.89, 18.38, 11.51. **EA** Calcd for C_28_H_44_Cl_2_O_2_S_2_Si_2_: C, 55.69; H, 7.34. Found: C, 55.79; H, 7.41.

### Single-Crystal X-Ray Structure Analysis of ClTn

A single-crystal of **ClTn** suitable for X-ray structure analysis was obtained by a liquid-liquid diffusion of CHCl_3_/MeOH solution. Data were collected on a Rigaku XtaLab P200 diffractometer with graphite monochromated MoKα radiation (*λ* = 0.71075 Å) in the *ω*-scan mode. The crystals were cooled by a stream of cold N_2_ gas. Collection, indexing, peak integration, cell refinement, and scaling of the diffraction data were performed using the CrystalClear software (Rigaku). The structures were solved by direct methods (SIR97) and refined by full-matrix least-square refinement on *F* ^2^ (SHELXL2014). The non-hydrogen atoms were refined anisotropically. All hydrogen atoms were placed on the calculated positions and refined using the riding model. The crystallographic data have been deposited at the Cambridge Crystallographic Data Centre (CCDC) under deposition number CCDC-2119698, and can be obtained free of charge via www.ccdc.cam.ac.uk/data_request/cif.

### Preparing and Characterizing ClTn-G and ClTn-Y

Typical procedure: to a stirred solution of **ClTn** (70.2 mg) in CHCl_3_ (4 ml) was added dropwise MeOH (30 ml). The precipitate was collected by vacuum filtration and washed with MeOH to obtain **ClTn-G**, which was then uniformly ground for 1 hour with an agate mortar and pestle to give **ClTn-Y**. The materials were characterized at room temperature in air. The steady-state photoluminescence (PL) spectra of **ClTn-G** and **ClTn-Y** were acquired using a JASCO FP-8200 spectrofluorometer with an L37 sharp-cut filter (HOYA, long pass, >370 nm) and a U340 band-pass filter (HOYA). Powder X-ray diffraction (PXRD) patterns were collected on a Rigaku MiniFlex 600 diffractometer with CuKα radiation (*λ* = 1.5418 Å). The PL quantum yields of **ClTn-G** and **ClTn-Y** were determined by the absolute method using a Hamamatsu photonics C11347-01 spectrometer augmented with an integrating sphere while excited at (*λ*_ex_) 368 nm. The PL decay curves were acquired with a HORIBA DeltaFlex multichannel scaling system using a DeltaDiode for excitation (368 nm). The PL intensity decay curves were recorded at 520 nm for **ClTn-G** and 560 nm for **ClTn-Y**. The area-weighted average lifetimes *τ* were determined with EzTime software (HORIBA) using a single-exponential fit for **ClTn-G** and a double-exponential fit for **ClTn-Y**.

### Evaluating Reversibility and Sensitivity

To evaluate reversibility of mechano/thermochromism and heat-induced recovery, **ClTn-G** was placed between two quartz plates and rubbed for 1 min, after which the PL spectrum was acquired (*λ*_ex_ = 320 nm). The plates were placed on a preheated copper plate and heated on a hot plate (IKA C-MAG HS 7) for 1 h, with the temperature of the copper plate maintained at 138–143°C. The sample was cooled to room temperature while on the plate (with the heater turned off), after which the PL spectrum was acquired (*λ*_ex_ = 320 nm). This treatment protocol was repeated four times to test repeatability. PL spectral change was evaluated as the color change ratio: First, the normalized difference in intensity *D* = (*I*_520_ – *I*_560_)/(*I*_520_ + *I*_560_) was determined for each spectrum to provide the relative intensity of the skew/TP emission, where *I*_x_ is the intensity at x nm. The color change ratio was then calculated as (*D* – *D*_G_)/(*D*_Y_ – *D*_G_) × 100 (%), where *D*_G_ and *D*_Y_ are the *D* values for the **ClTn-G** and **ClTn-Y**, respectively. All photographic images were acquired using a SONY NEX-5N camera while irradiated with a hand-held UV light (365 nm). Temperature was controlled in a similar manner to evaluate sensitivity toward thermal stimulation. The recovery ratio is defined as (*D* – *D*_h0_)/(*D*_G_ – *D*_h0_) × 100 (%), where *D*_h0_ is the *D* value for sample h0 (**ClTn-G** rubbed for 1 min between two quartz plates). The color change and recovery ratios of **BrTn** were determined using *I*_570_ instead of *I*_560_.

### Constructing the *Φ*_P_-Weighted PL Spectrum of ClTn-Y

The spectrum of **ClTn-G**, which is purely derived from the skew conformer, was subtracted from that of **ClTn-Y** after normalizing the intensity at *λ*_max_ (the emission maximum) of **ClTn-G**. The difference spectrum corresponds to the pure PL spectrum of the TP conformer. The difference spectrum and the spectrum of **ClTn-G** were multiplied by *α*_Y_ = [*Φ*_p_ of **BrTn-Y**]/[*Φ*_p_ of **ClTn-Y**] and *α*_G_ = [*Φ*_p_ of **BrTn-G**]/[*Φ*_p_ of **ClTn-G**], respectively, and summed to construct the *Φ*_p_-weighted PL spectrum of **ClTn-Y**.

## Results and Discussion

**FIGURE 2:**
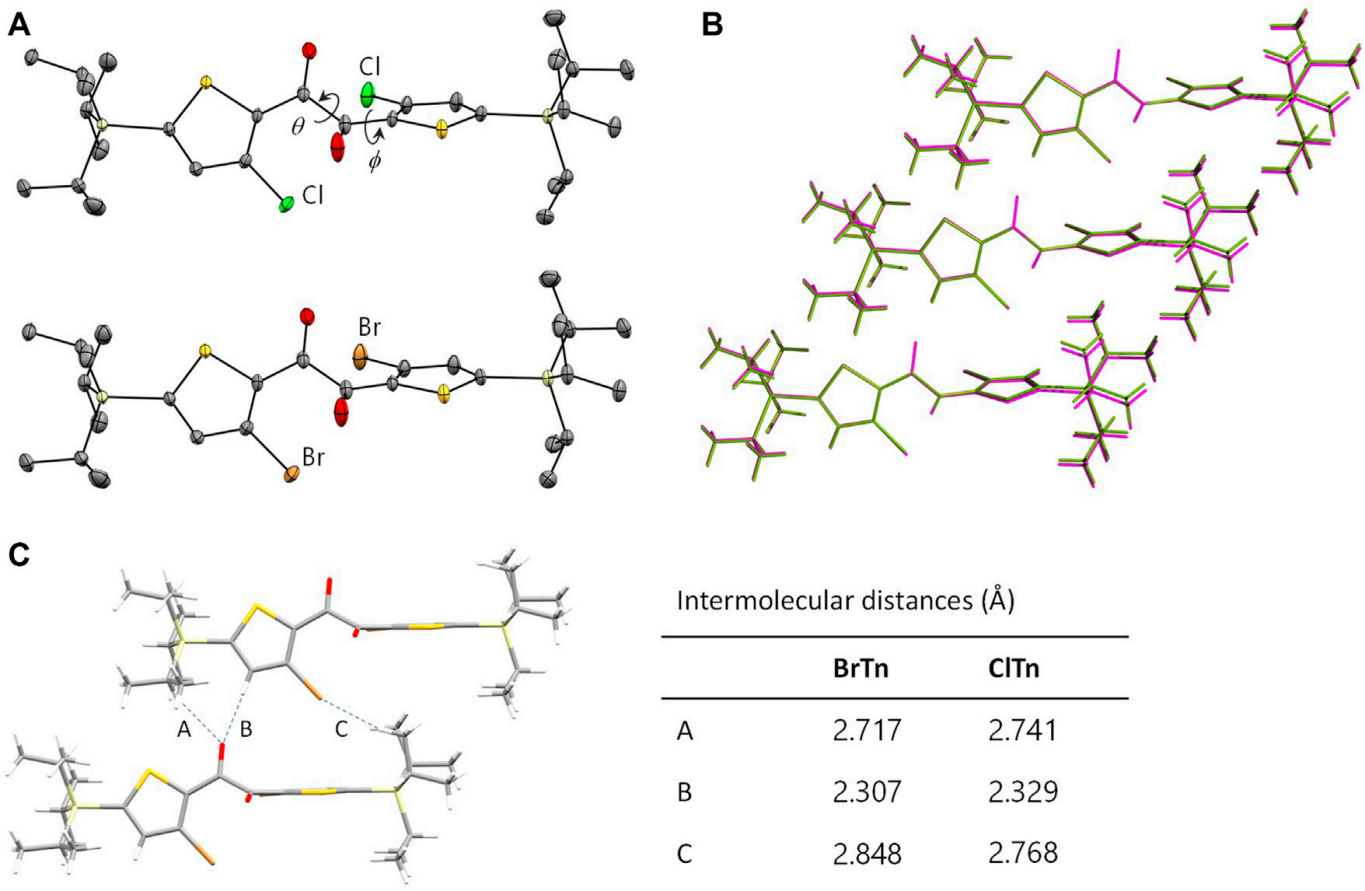
**(A)** ORTEP drawings of the X-ray crystal structures of **ClTn** **(top)** and **BrTn** **(bottom)**. Hydrogen atoms are omitted for clarity and the thermal ellipsoids are set at the 50% probability level. **(B)** Overlaying three successive molecules in the crystals of **ClTn** (green) and **BrTn** (magenta). **(C)** Intermolecular short contact distances (Å) found in the crystal structures of **BrTn** and **ClTn**.

Diketone **ClTn** was synthesized by reacting **BrTn** with CuCl. The X-ray structure of a single-crystal of **ClTn** revealed that it is isostructural to that of **BrTn** (CCDC), with superimposable molecular geometries, the same space group, and very similar lattice constants (Figure 2 and Table 1). The conformation of aromatic 1,2-diketones is well represented by two torsion angles: the vicinal-dicarbonyl torsion angle *θ* and the thiophene–carbonyl torsion angle *ϕ* (Mukai et al., 1992; Singh et al., 2002). A comparison of these angles in **ClTn** and **BrTn** highlights their almost identical crystal conformations (*θ*: 114.0(1) vs. 109.5(2)°; *ϕ*: –19.1(2) vs. –17.2(3)°). In addition, the diketones have three kinds of intermolecular interactions (a total of 12 interactions from one molecule), which are also comparable in both systems due to their identical crystal packing (Figure 2C). Hence, these isostructural crystals provide both conformationally and environmentally consistent systems that are ideal for investigating structure–property relationships.

**TABLE 1:** Crystallographic data for **ClTn** and **BrTn**.

	Space group	*a*/Å	*b*/Å	*c*/Å	*α*/o	*β*/o	*γ*/o	*θ*/o	*ϕ*/o
**ClTn**	*C*2/*c*	12.0873(13)	9.4966(10)	27.677(3)	90	91.255(3)	90	114.0(1)	–19.1(2)
**BrTn**	*C*2/*c*	12.0795(9)	9.5698(6)	27.9836(17)	90	92.019(7)	90	109.5(2)	–17.2(3)

**FIGURE 3:**
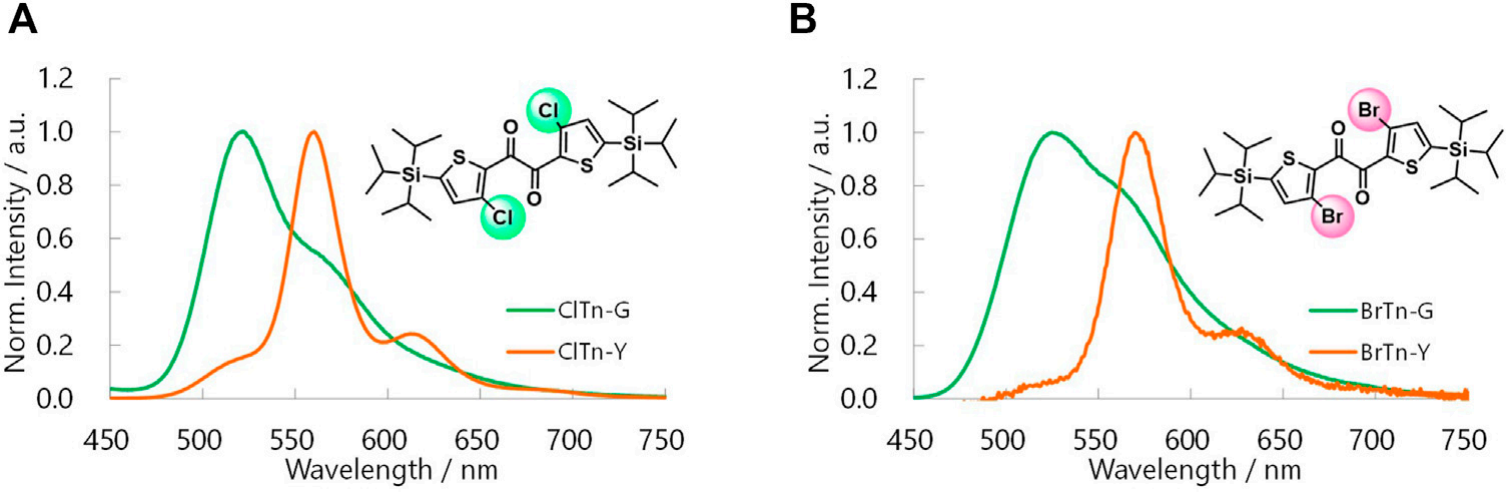
Steady-state PL spectra of **(A) ClTn** and **(B) BrTn** at room temperature in air. *λ*_ex_ = 320 and 360 nm for the G and Y phase samples, respectively.

**FIGURE 4:**
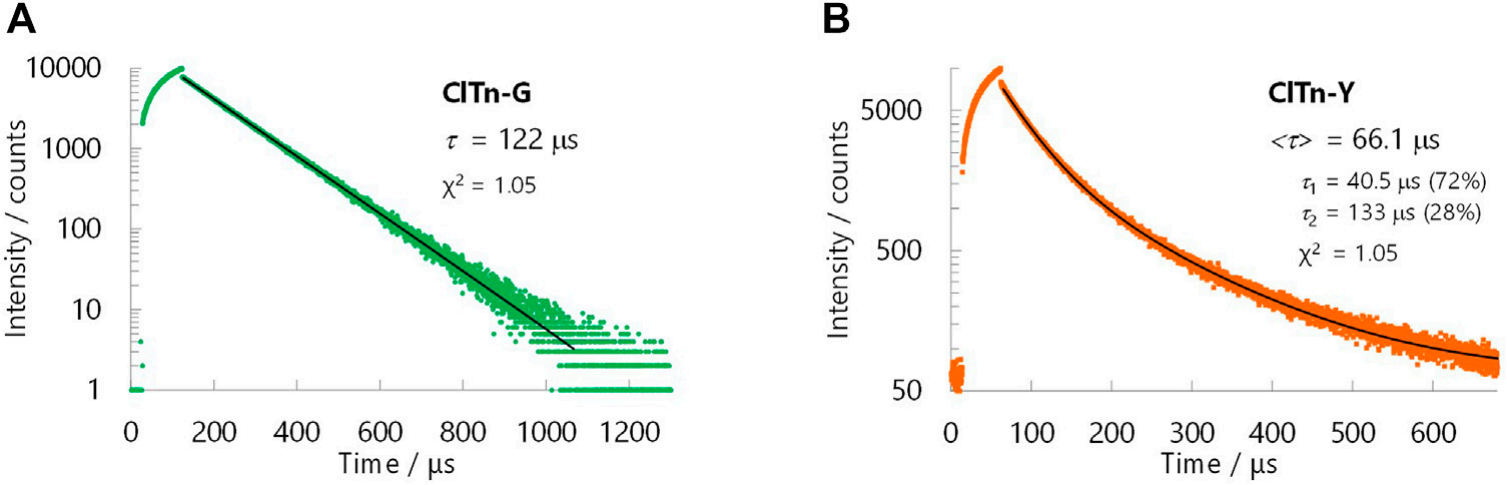
PL decay curves for **(A) ClTn-G** and **(B) ClTn-Y** at room temperature in air. The black lines are fitted curves. PL intensities were recorded at 520 nm for **ClTn-G** and 560 nm for **ClTn-Y** (*λ*_ex_ = 368 nm). The bracketed lifetime <*τ*> is the area-weighted lifetime.

**FIGURE 5:**
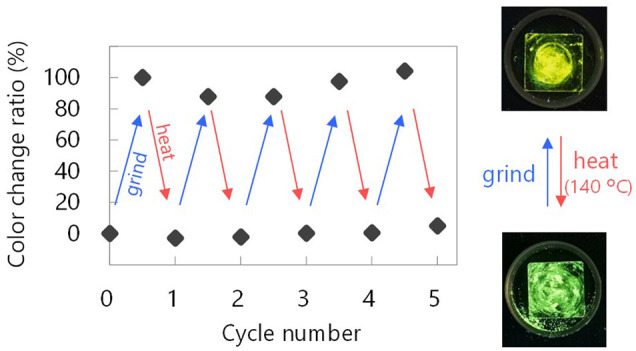
Showing reversibility of the mechano/thermoresponsive RTP chromism of **ClTn**. Photographic images were acquired under UV light (365 nm). See Experimental Section for definition of the color change ratio.

**FIGURE 6:**
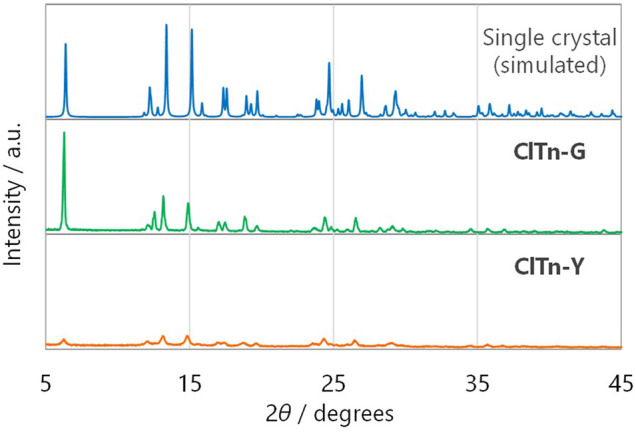
Powder X-ray diffraction patterns of (from **top** to **bottom**) single-crystals of **ClTn** (simulated), **ClTn-G**, and **ClTn-Y**.

**ClTn** was found to exhibit RTP-to-RTP mechanochromism in a qualitatively similar manner to **BrTn** (Tani et al., 2019); the RTP color changed from green (G-phase) to yellow (Y-phase) upon grinding. To investigate these photophysical properties in detail, we prepared samples of **ClTn-G** and **ClTn-Y**; **ClTn-G** is a crystalline powder reprecipitated from CHCl_3_/MeOH, while **ClTn-Y** was prepared by uniformly grinding **ClTn-G** using an agate mortar and a pestle (see Experimental Section for details). The photoluminescence (PL) emission maximum of **ClTn-Y** (*λ*_PL_ = 560 nm) was observed to be redshifted by ∼40 nm compared to that of **ClTn-G** (*λ*_PL_ = 522 nm) (Figure 3A). By comparing the behavior of **BrTn** with that of **ClTn** (Figure 3B), (Tani et al., 2019) we conclude that the PL spectrum of **ClTn-Y** consists of an emerging emission from the TP conformer and a remaining small emission from the skew conformer (vide infra). The PL lifetimes of **ClTn-G** and **ClTn-Y** were determined to be 66 and 122 μs, respectively, without any nanosecond-order decay component, confirming that these are phosphorescence emissions (Figure 4). Moreover, the yellow RTP of **ClTn-Y** returned to green upon heating (Figure 5); this color-change cycle was repeated for five-times, thereby demonstrating reversible mechano/thermoresponsiveness. The observed RTP mechanochromism is based on crystal amorphization, because the PXRD pattern of **ClTn-G** reveals sharp diffraction peaks, while that of **ClTn-Y** shows peak broadening (Figure 6). Overall, the RTP-to-RTP mechanochromism of **BrTn** was well reproduced by **ClTn**; however, close inspection revealed notable differences in stimulus-responsiveness that are compared and mechanistically rationalized below.

**FIGURE 7:**
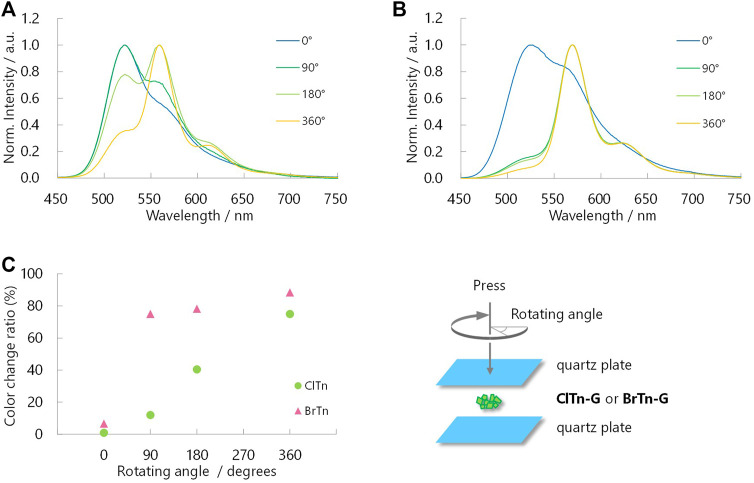
Normalized PL spectra of **(A) ClTn-G** and **(B) BrTn-G** upon mechanical stimulation (*λ*_ex_ = 320 nm). Angles in the legends are the rotating angles as depicted in the bottom right. **(C)** Sensitivity of RTP color to mechanical stimulation. See Experimental Section for definition of the color change ratio.

Despite its isostructural nature, the RTP color of **ClTn** responds to mechanical stimulus more slowly than that of **BrTn**. For comparison, crystalline powder (**ClTn-G** or **BrTn-G**) was placed between two quartz plates, ground by rotating the upper plate while being pressed, with PL spectra acquired at rotating angles of 0, 90, 180, and 360° (Figure 7). The RTP color of **ClTn** changed gradually; the relative intensity of the emission from the TP conformer (*λ*_PL_ = 560 nm) continuously increased with the application of the mechanical stimulus. On the other hand, the TP emission of **BrTn** emerged rapidly; its RTP color changed dramatically during the first quarter turn and remained almost unchanged thereafter (Figure 7C). These results may seem to suggest that **BrTn-G** is more easily amorphized than **ClTn-G**; however, we note that what responds faster in **BrTn** is the RTP color, for which the ease of amorphization is just one of the possible factors. Evaluating/monitoring the rate of amorphization (loss of crystallinity) during mechanical stimulation is difficult because the preferred orientation may also affect the PXRD peak, the full-width at half-maximum (FWHM) of which is a measure of crystallinity, and can be alleviated by mechanical stimulation.

**FIGURE 8:**
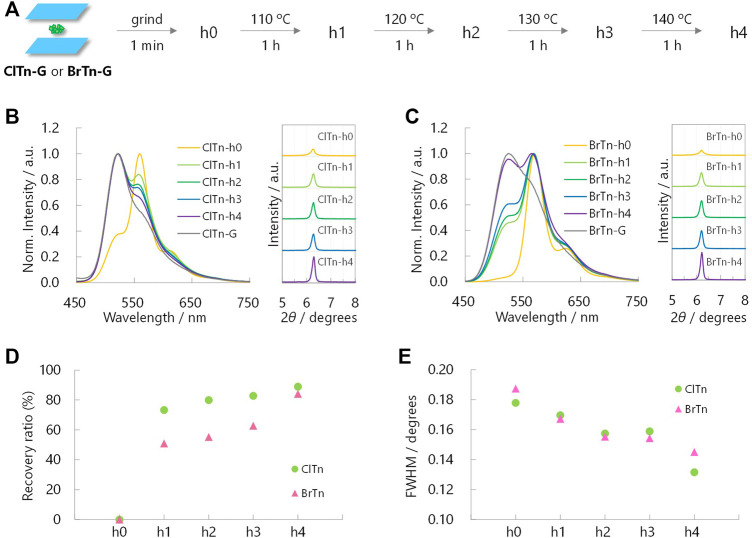
**(A)** Sample preparation for heat-induced-recovery experiments. **(B, C)** Normalized PL spectra **(left,** *λ*_ex_ = 320 nm) and corresponding PXRD profiles **(right)** of **(B) ClTn-Y** and **(C) BrTn-Y** upon heating. **(D)** Sensitivity of the RTP color and **(E)** FWHMs of the PXRD peaks at 2 *θ* = ∼6° toward the thermal stimulation. See Experimental Section for definition of the recovery ratio.

Monitoring the heat-induced recovery from the Y phase to the G phase is expected to provide a better understanding of the crystallinity–RTP color relationship, as heating does not disturb the orientation of the powder. With this in mind, PL spectra and PXRD profiles were acquired after heating the Y phase samples (h0) at the temperatures and times indicated in Figures 8A–C. In contrast to the mechanical response observed for **ClTn**, its PL spectrum responded faster to temperature than the spectrum of **BrTn**. More interestingly, **ClTn** and **BrTn** exhibited the same crystallinity response; the FWHMs of the PXRD peaks at 2*θ* = 6° were observed to decrease at the same rate (Figures 8D,E). These results indicate that, while the crystallinity of the powder is important for determining the RTP color, the stimulus-responsiveness of RTP color is not associated with ease of amorphization.

**FIGURE 9:**
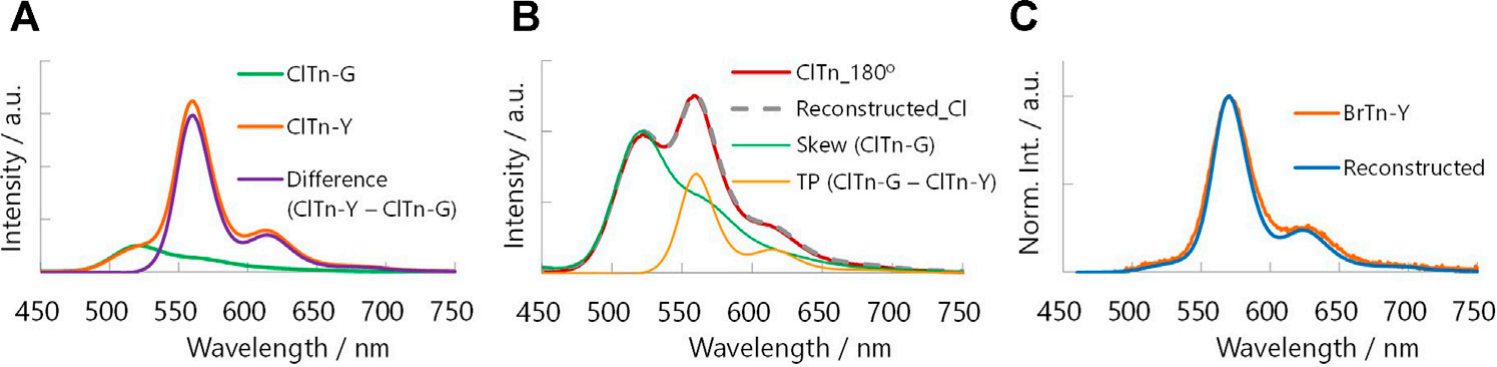
**(A)** Deconvolution of the PL spectrum of **ClTn-Y** (orange) by subtracting that of **ClTn-G** (green). **(B)** Reconstructing the PL spectrum of partially ground **ClTn** (red, 180° in Figure 7A) from those of the skew (green) and TP conformer (orange). **(C)** Reconstructed *Φ*_p_-weighted PL spectrum of **ClTn-Y** (blue, shifted by –10 nm) and the PL spectrum of **BrTn-Y** (orange).

**FIGURE 10:**
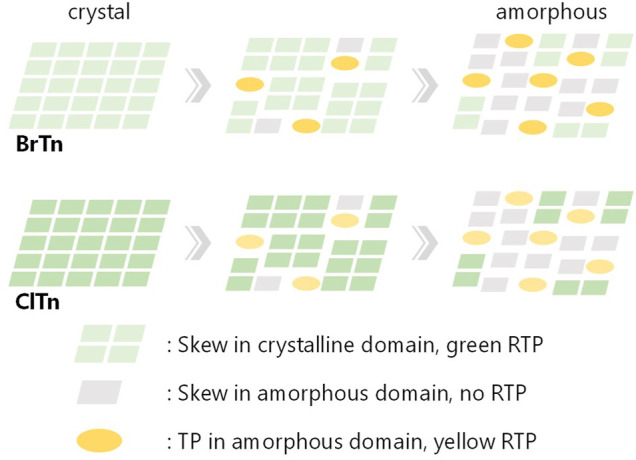
Schematic representation of the proposed mechanism for different mechanoresponse of RTP color, describing the change in conformation and RTP color along amorphization. The relative color depth represents relative PL efficiency.

We hypothesize that the difference in the stimulus-responsiveness of the RTP color originates from a conformation-dependent heavy atom effect. To test this hypothesis, we determined the phosphorescence quantum yields *Φ*_p_ of each compound/phase and constructed the *Φ*_p_-weighted PL spectrum of **ClTn-Y**. As expected from the weaker heavy atom effect of Cl compared to Br, the *Φ*_p_ of **ClTn** is smaller than that of **BrTn** (Table 2). Interestingly, the amorphous Y phase exhibited a larger quantum yield ratio (*α* = [*Φ*_p_ of **BrTn**]/[*Φ*_p_ of **ClTn**]) than the crystalline G phase, implying that the extent of the heavy atom effect depends on conformation. Next, we subtracted the PL spectrum of **ClTn-G**, which is purely derived from the skew conformer, from that of **ClTn-Y** (Figure 9A). The difference spectrum corresponds to the pure PL spectrum of the TP conformer. Indeed, other PL spectra of **ClTn** with partial amorphization (e.g., 180° in Figure 7A) can be reconstructed from the obtained spectra of the skew and TP conformers (Figure 9B), which indicates that the PL spectra are composed of the two emissions. Finally, we corrected the PL spectrum of **ClTn-Y** by multiplying the spectra of each conformer by *α* and then recombining them. The *Φ*_p_-weighted PL spectrum of **ClTn-Y** (Figure 9C, blue trace) obtained in this manner matches the PL spectrum of **BrTn-Y**; therefore, we concluded that the conformational composition of amorphous **ClTn-Y** is similar to that of **BrTn-Y**, and that the differences in spectral shape are attributable to the difference in PL efficiency. A proposed mechanism for the different stimulus-responsivenesses of RTP color is described in Figure 10. Even though the ease of amorphization is similar as expected for the isostructural crystals, different PL efficiency (represented by color depth) makes the RTP color response different. Upon amorphization, the PL quantum yields are increased in **BrTn-G/Y** (3.9/10%) while slightly decreased in **ClTn-G/Y** (1.7/1.4%) (Table 2). Thus, the mechanoresponse in the RTP color is different. We emphasize that, in the present system, isostructural crystals exhibited a similar amorphization behavior, enabling the stimulus-responsiveness of the bulk solid to be modulated by tuning the molecular properties.

**TABLE 2:** Phosphorescence maxima, quantum yields, and lifetimes.

	*λ*_p_/nm	*Φ*_p_ (%)	τ/μs
**ClTn-G**	522	1.7	122
**BrTn-G**	527	3.9	103
**ClTn-Y**	560	1.4	66
**BrTn-Y**	571	10	51

## Conclusion

The RTP-to-RTP mechanochromism of a thienyl diketone was successfully modulated by Br-to-Cl halogen exchange. Modulating the molecular structure does not disturb the crystal structure, with both diketones forming isostructural crystals. The stimuli-responsiveness of the crystallinity is also retained, as evidenced by PXRD peak widths. In contrast, relative RTP efficiency is affected, which is ascribable to a conformation-dependent heavy-atom effect. Consequently, we were able to successfully modulate the stimulus-responsiveness of RTP color. We are currently investigating the conformation-dependent heavy atom effect, which will be reported in due course.

## Data Availability

The datasets presented in this study can be found in online repositories. The names of the repository is Cambridge Crystallographic Data Centre. Following are the accession numbers CCDC-2119698 CCDC-1906440.
