# Supplementary material for: Modulating Room-Temperature Phosphorescence-To-Phosphorescence Mechanochromism by Halogen Exchange
Source: Front Chem. 2022 Jan 13;9:812593. doi: 10.3389/fchem.2021.812593 (PMC8793743; doi:10.3389/fchem.2021.812593)
Supplement: Supplementary file 1 [file DataSheet1.PDF]

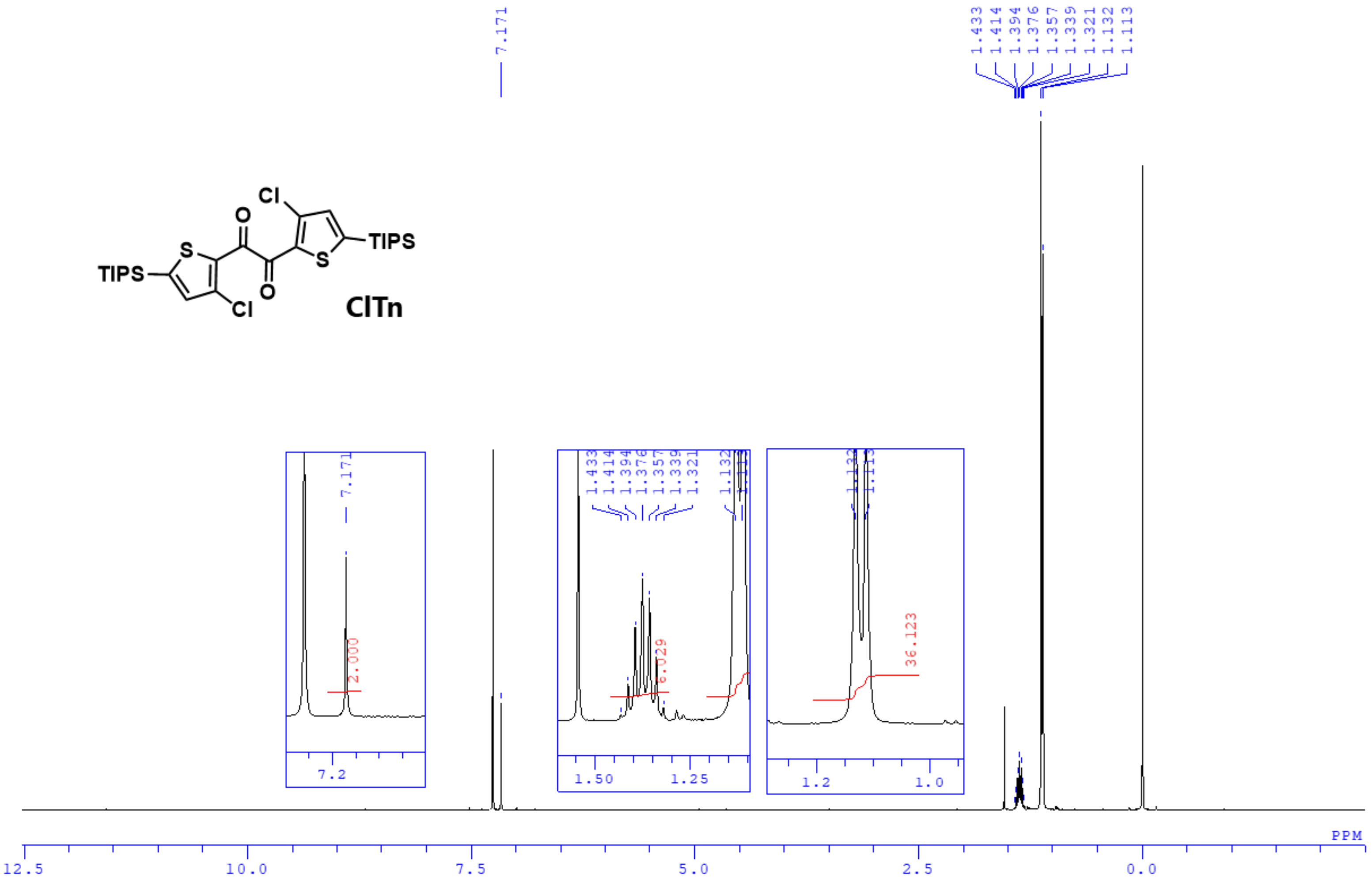

Figure S1. ^{1}H NMR spectrum of **ClTn** (400 MHz, $CDCl_3$).

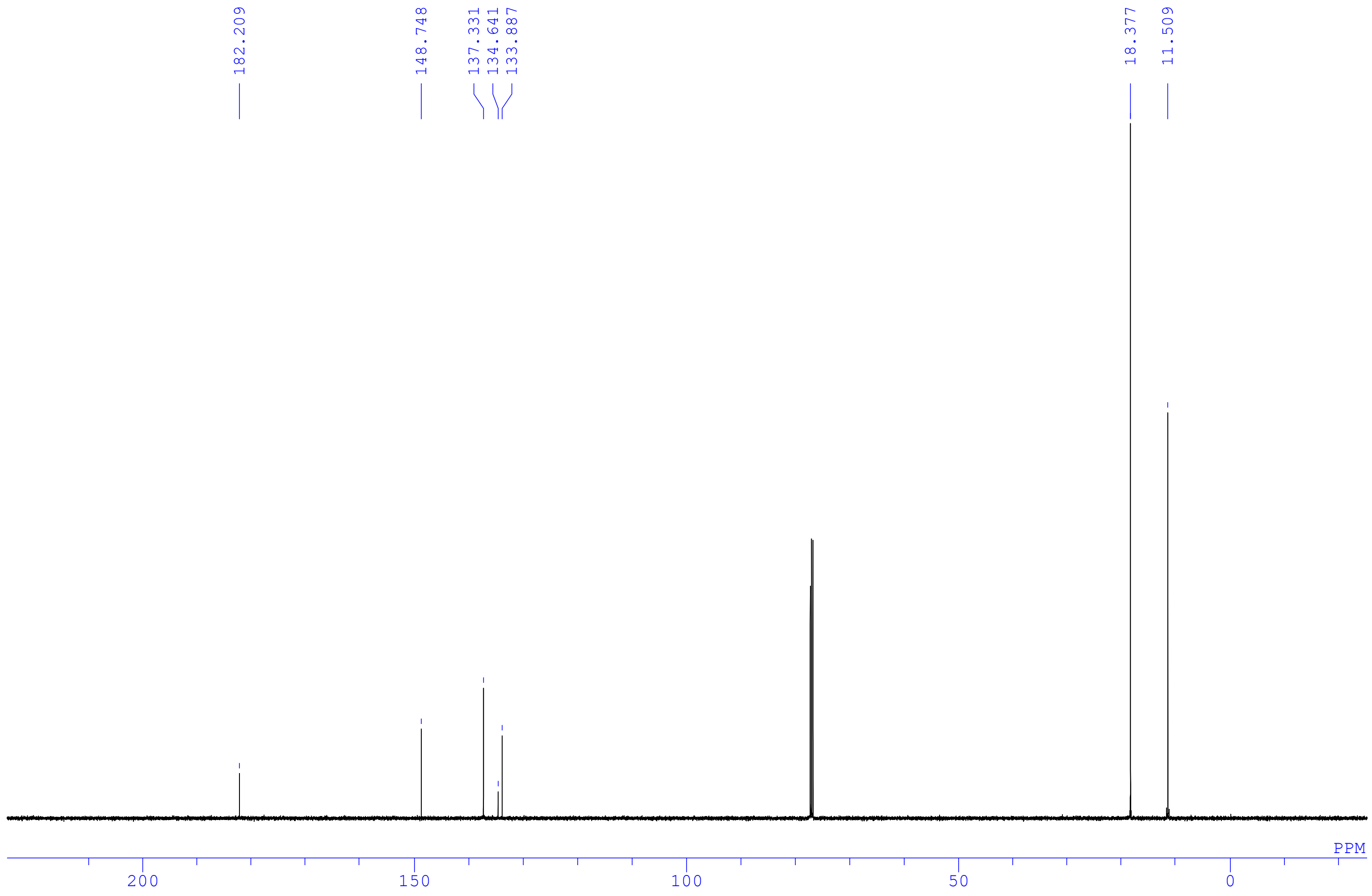

Figure S2. $^{13}C\{^{1}H\}$ NMR spectrum of **ClTn** (100 MHz, $CDCl_3$).
